# Deciphering downstream gene targets of PI3K/mTOR/p70S6K pathway in breast cancer

**DOI:** 10.1186/1471-2164-9-348

**Published:** 2008-07-24

**Authors:** Henna Heinonen, Anni Nieminen, Matti Saarela, Anne Kallioniemi, Juha Klefström, Sampsa Hautaniemi, Outi Monni

**Affiliations:** 1 Institute of Biomedicine and Biomedicum Biochip Center, Genome-Scale Biology Research Program, University of Helsinki, Finland; 2 Cancer Cell Circuitry Laboratory, Institute of Biomedicine, Genome-Scale Biology Research Program, University of Helsinki, Finland; 3 Institute of Software Systems, Tampere University of Technology, Finland; 4 Laboratory of Cancer Genetics, Institute of Medical Technology, University of Tampere and Tampere University Hospital, Finland; 5 Computational Systems Biology Laboratory, Institute of Biomedicine, Genome-Scale Biology Research Program, University of Helsinki, Finland

## Abstract

**Background:**

The 70 kDa ribosomal protein S6 kinase (*RPS6KB1*), located at 17q23, is amplified and overexpressed in 10–30% of primary breast cancers and breast cancer cell lines. p70S6K is a serine/threonine kinase regulated by PI3K/mTOR pathway, which plays a crucial role in control of cell cycle, growth and survival. Our aim was to determine p70S6K and PI3K/mTOR/p70S6K pathway dependent gene expression profiles by microarrays using five breast cancer cell lines with predefined gene copy number and gene expression alterations. The p70S6K dependent profiles were determined by siRNA silencing of *RPS6KB1 *in two breast cancer cell lines overexpressing p70S6K. These profiles were further correlated with gene expression alterations caused by inhibition of PI3K/mTOR pathway with PI3K inhibitor Ly294002 or mTOR inhibitor rapamycin.

**Results:**

Altogether, the silencing of p70S6K altered the expression of 109 and 173 genes in two breast cancer cell lines and 67 genes were altered in both cell lines in addition to *RPS6KB1*. Furthermore, 17 genes including *VTCN1 *and *CDKN2B *showed overlap with genes differentially expressed after PI3K or mTOR inhibition. The gene expression signatures responsive to both PI3K/mTOR pathway and p70S6K inhibitions revealed previously unidentified genes suggesting novel downstream targets for PI3K/mTOR/p70S6K pathway.

**Conclusion:**

Since p70S6K overexpression is associated with aggressive disease and poor prognosis of breast cancer patients, the potential downstream targets of p70S6K and the whole PI3K/mTOR/p70S6K pathway identified in our study may have diagnostic value.

## Background

The 70 kDa ribosomal protein S6 kinase (p70S6K) is a mitogen-activated serine/threonine kinase that has a critical role in control of cell cycle, growth and survival. p70S6K is encoded by *RPS6KB1*, which is located at 17q23 and is amplified and overexpressed in 10–30% of breast cancer cell lines and primary breast cancers [1-4]. The overexpression of p70S6K is associated with aggressive disease and poor prognosis of breast cancer patients [2]. p70S6 kinase is located downstream of PI3K/AKT/mTOR pathway, which is activated by HER2 receptors, insulin-like growth factor receptor and estrogen receptor in breast cancer [5]. p70S6K itself is activated by 3-phosphoinositide-dependent protein kinase 1 (PDK-1) and mammalian target of rapamycin (mTOR) kinase. p70S6 kinase regulates protein synthesis by activating 40S ribosomal protein S6, leading to an increased rate of translation of the class of 5'TOP (5' terminal oligopyrimide) mRNA transcripts. These transcripts encode critical components of the cellular translational machinery, thus promoting protein synthesis [6,7]. Additionally, p70S6K has a crucial role in cell growth by regulating cell size and progression of cell cycle [8-10]. Recently, p70S6K has been reported to inactivate the pro-apoptotic molecule BAD by phosphorylation, thereby also promoting cell survival [11].

PI3K/AKT/mTOR pathway is often activated in cancer due to genetic alterations of the genes implicated in this pathway. For example, *PIK3CA*, *PTEN*, *TSC1/2*, *HER2*, *AKT*, and *PDPK1 *have been found to be frequently mutated or amplified in cancer and thereby PI3K/AKT/mTOR pathway is an attractive target for therapeutics. In clinical trials, there are a number of drugs that target proteins involved in this pathway [12,13]. For example, flavonoid derivative Ly294002 is a PI3K inhibitor that acts in the ATP-binding site of PI3K enzyme and targets the PI3K/AKT axis [14]. Rapamycin is an immunosuppressant and a potential clinical drug that inhibits mTOR by binding to the phosphatidic acid-binding site required for mTOR activation [15,16]. Thus, mTOR cannot phosphorylate p70S6 kinase resulting in G1 arrest of the cell cycle and suppression of protein synthesis. Despite the fact that PI3K/AKT/mTOR pathway contains many putative therapeutic targets, the clinical trials with the pathway-specific drugs have not been as promising as previously thought. This might be due to the cross-talk of PI3K/AKT/mTOR pathway with multiple other signalling pathways leading to multiple sites of regulation. Similarly, the diversity of genetic aberrations activating this pathway is likely to cause differences in drug responses.

Our aim was to identify genes that are transcriptionally altered due to PI3K/mTOR/p70S6K pathway inhibition in breast cancer cells using RNAi and small molecule inhibitors. p70S6K encoded by *RPS6KB1 *was knocked down using three different siRNAs in BT-474 and MCF-7 breast cancer cell lines, since these cell lines show high-level amplification and overexpression of *RPS6KB1*. Ly294002 and rapamycin are known to target PI3K/mTOR pathway upstream of p70S6K. Therefore, breast cancer cell lines BT-474, MCF-7, MDA-361, MDA-436 and SK-BR-3 were treated with these inhibitors to compare transcriptional signatures responsive to both *RPS6KB1 *and PI3K/mTOR pathway inhibitions. Our results show for the first time the genome-wide transcriptional consequences of PI3K/mTOR pathway and *RPS6KB1 *inhibitions in breast cancer, suggesting novel downstream targets for PI3K/mTOR pathway and p70S6 kinase.

## Results

### p70S6K suppression induces specific gene expression alterations

**Figure 1:**
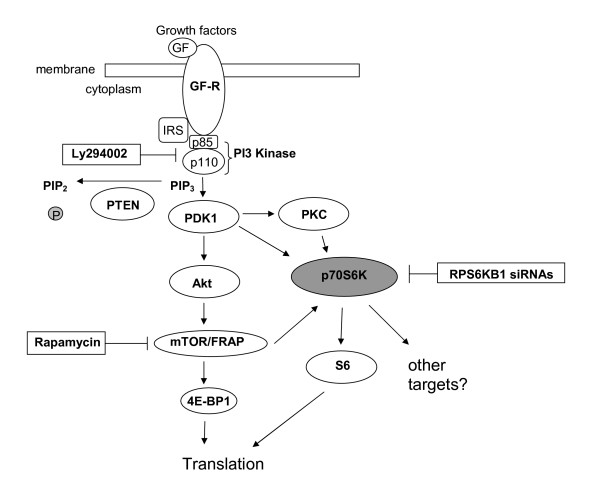
**Interfering the PI3K/mTOR/p70S6K signaling pathway**. Schematic representation of the PI3K/mTOR/p70S6K pathway illustrates that p70S6K is located downstream of PI3K and mTOR. In this study, PI3K was inhibited with Ly294002, mTOR with rapamycin and p70S6K with siRNAs targeting *RPS6KB1*.

**Figure 2:**
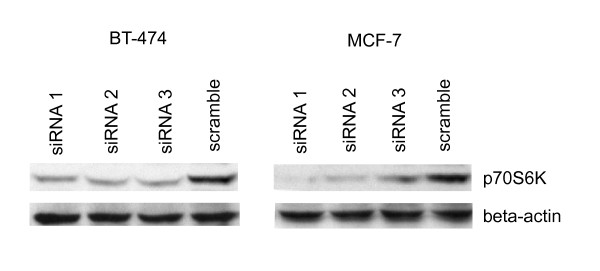
**Protein-level validation of p70S6K suppression after *RPS6KB1 *siRNA treatments in breast cancer cell lines**. BT-474 and MCF-7 cells were treated with three siRNAs against *RPS6KB1 *and one scramble oligo for 72 hours and the protein expressions were detected by Western immunoblotting. The p70S6K expression was significantly downregulated in siRNA-transfected samples when compared to the scramble oligo transfected samples. Beta-actin was used as a loading control.

To identify downstream targets of p70S6K in breast cancer cells, we first examined gene expression alterations in *RPS6KB1*-suppressed BT-474 and MCF-7 breast cancer cell lines that normally show high-level expression of p70S6K. We used three different siRNAs to knock-down the expression of *RPS6KB1 *(Figure 1). Based on the microarray analyses, the signal log10 ratio with siRNA 1 was -0.5, resulting in 70% relative downregulation of *RPS6KB1 *mRNA, whereas with *RPS6KB1 *siRNAs 2 and 3 log10 ratios were -0.3 – -0.5 with different probes representing *RPS6KB1*, indicating 50–70% relative suppression with these two siRNAs. The signal log10 ratios of all the genes representing their mRNA expression levels are available at CanGEM (please see Availability & requirements for more information). The *RPS6KB1 *knock-down also caused significant decrease in p70S6K protein expression after 72 hours in both cell lines (Figure 2).

The microarray analyses showed that altogether 109 and 173 genes displayed over two-fold difference with at least two different *RPS6KB1 *siRNAs in BT-474 and MCF-7 breast cancer cell lines, respectively. Of these differentially expressed genes, 68 genes (39–62%) were commonly down- or up-regulated in both cell lines including *RPS6KB1*, *ABL1*, *PPP1R12B*, *PRKCQ*, and *STK32B *[see Additional File 1]. Sixty-nine genes (63%) were down- or up-regulated in BT-474 with all three siRNAs, whereas ninety-one genes (53%) were differentially expressed with all siRNAs in MCF-7. From these, 45 genes were differentially expressed with all siRNAs in both of the breast cancer cell lines [see Additional File 1].

### Rapamycin and Ly294002 block Thr389 phosphorylation of p70S6K

**Figure 3:**
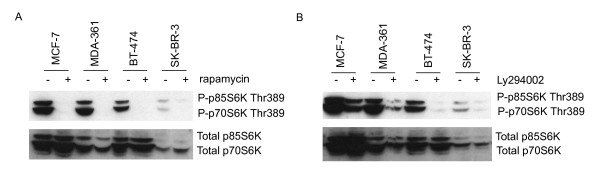
**Effects of rapamycin and Ly294002 treatments on phosphorylation of p70S6K^Thr389 ^in breast cancer cell lines**. The cells were treated with A) 100 nM rapamycin and B) 50 μM Ly294002 for 24 hours and the phosphorylation status of p70S6K Thr389 was detected by Western immunoblotting. The plus (+) sign indicates the inhibitor-treated samples and the minus (-) sign the non-treated samples. Both rapamycin and Ly294002 blocked Thr389 phosphorylation of p70S6K. The total p70S6K expression was used as a reference.

To further study the downstream targets of PI3K/mTOR pathway, five breast cancer cell lines BT-474, MCF-7, MDA-361, MDA-436 and SK-BR-3 were treated with PI3K inhibitor Ly294002 and mTOR inhibitor rapamycin (Figure 1). We first evaluated the phosphorylation status of p70S6K after inhibitor treatments by Western blotting. Previously, we have shown [2,3] that MCF-7, BT-474 and MDA-361 breast cancer cell lines have p70S6K amplification and protein overexpression, whereas SK-BR-3 show normal copy number and protein expression levels of p70S6K. After the inhibitor treatments with Ly294002 and rapamycin, significantly lower or no protein expression of Thr389-phosphorylated p70S6K was detected by Western blotting in all the rapamycin- and Ly294002-treated breast cancer cell lines as compared to non-treated samples (Figure 3A–B). Our results indicate that rapamycin and Ly294002 blocked phosphorylation of Thr389, one of the amino acids essential for p70S6K activation. The total p70S6K levels were higher in MCF-7, MDA-361 and BT-474 breast cancer cell lines as compared to that of SK-BR-3 (Figure 3A–B). In MDA-436 breast cancer cell line, the expression of total p70S6K was hardly detectable (data not shown).

### Ly294002 and rapamycin treatments induce similar gene expression profiles with different biological outcomes

**Figure 4:**
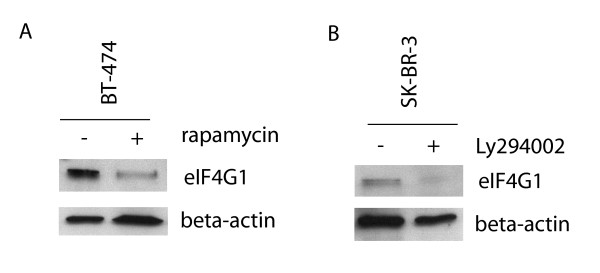
**Protein-level downregulation of eIF4G1 in rapamycin-treated BT-474 cells and in Ly294002-treated SK-BR-3 cells**. The breast cancer cells were treated with 100 nM rapamycin and 50 μM Ly294002 for 24 hours and the protein expression of eIF4G1 was detected by Western immunoblotting. The plus (+) sign indicates the inhibitor-treated sample and the minus (-) sign the non-treated sample. A) Rapamycin and B) Ly294002 downregulated eIF4G1 expression at protein level in BT-474 and SK-BR-3 cells, respectively. Beta-actin was used as a loading control.

**Figure 5:**
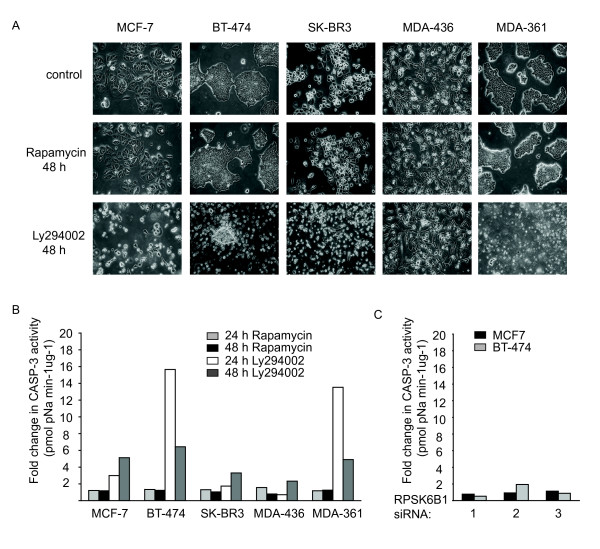
**Effects of rapamycin, Ly294002 and *RPS6KB1 *siRNA treatments on apoptosis of breast cancer cell lines**. A) The cells were treated with 100 nM rapamycin and 50 μM Ly294002 for 48 hours and the cell morphology was evaluated under the light microscope. B) Caspase-3 activity assay of rapamycin- and Ly294002-treated breast cancer cell lines. C) Caspase-3 activity assay of *RPS6KB1 *siRNA-treated breast cancer cell lines. The values are averages from two or three independent experiments. Ly294002 induced apoptosis in BT-474 and MDA-361 breast cancer cells, whereas rapamycin had no effect. Similarly, knock-down of *RPS6KB1 *did not have any effect on apoptosis in BT-474 and MCF-7 breast cancer cells.

We then studied gene expression alterations caused by inhibition of PI3K/mTOR pathway by PI3K inhibitor Ly294002 and mTOR inhibitor rapamycin. SOM analysis was performed in order to detect the overall gene expression alterations in five inhibitor-treated breast cancer cell lines. In 24 h and 48 h time point treatments, 537 and 577 genes were differentially expressed in Ly294002-treated cells and 538 and 523 genes were differentially expressed in rapamycin-treated cells, respectively [see Additional File 2]. Altogether, 38% of the differentially expressed genes were altered in both the Ly294002 and rapamycin treatments at 24 h time point. Additionally, several genes associated with PI3K/mTOR pathway were altered at the transcriptional level [see Additional File 2]. For example, *AKT1*, *FRAP1, EIF4E, EIF4G1, EIF4A1 *and *EIF4A2 *were transcriptionally altered in several inhibitor-treated cell lines but not in cell lines treated with siRNAs targeting *RPS6KB1 *[see Additional Files 1 and 2]. The protein expression of three of these genes, *AKT1*, *FRAP1 *and *EIF4G1 *(encoding AKT, mTOR and eIF4G1, respectively), were detected by Western immunoblotting and eIF4G1 showed downregulation at protein level by both rapamycin and Ly294002 treatments (Figure 4A–B). To evaluate the biological responses to PI3K/mTOR pathway inhibitors, cell cycle and apoptosis assays were performed for inhibitor-treated breast cancer cell lines. Ly294002 treatment caused apoptosis in BT-474 and MDA-361 (Figure 5A–B) and G1 arrest of the cell cycle in MCF-7 and SK-BR-3 breast cancer cell lines [see Additional File 3]. Rapamycin arrested cells in G1 phase in three cell lines (MCF-7, BT-474 and MDA-361) after 24 h or 48 h treatment [see Additional File 3] without inducing apoptosis in any of the cell lines (Figure 5A–B). The suppression of p70S6K with *RPS6KB1 *siRNAs did not induce apoptosis (Figure 5C).

### Genes responsive to both PI3K/mTOR pathway and p70S6K inhibitions reveal novel putative downstream targets of PI3K/mTOR/p70S6K pathway

We also compared the individual gene expression profiles between Ly294002- and rapamycin-treated and *RPS6KB1*-suppressed BT-474 and MCF-7 breast cancer cell lines to identify genes downstream of PI3K/mTOR/p70S6K pathway. Expression of 17 genes was altered in BT-474 and MCF-7 breast cancer cell lines in response to both PI3K or mTOR inhibition and p70S6K inhibition with at least two siRNAs (Table 1). In BT-474 cell line, these included 9 genes, e.g. *ARL11 *and *CDKN2B*. Also in MCF-7 cell line, 9 genes were differentially expressed after siRNA and inhibitor treatments including *VTCN1*, *SCD *and *RELB*. *ST3GAL6 *was differentially expressed in both cell lines. Altogether, the inhibition of PI3K and mTOR in BT-474 and MCF-7 cells by Ly294002 and rapamycin led to differential expression of a higher number of genes than with *RPS6KB1 *siRNAs [see Additional File 4]. The number of differentially expressed genes after Ly294002 treatment was 530, whereas after rapamycin treatment it was 117. The higher number of genes after Ly294002 treatment is somewhat expected since Ly294002 inhibition caused the most effective biological response (Figure 5, see Additional File 3). Knock-down of p70S6K caused differential expression of only 68 genes [see Additional Files 1 and 4] and no apoptosis was detected in BT-474 and MCF-7 cell lines (Figure 5).

**Table 1:** Genes responsive to PI3K, mTOR and p70S6K inhibitions in breast cancer cell lines.

**BT-474 breast cancer cell line, RPS6KB1-suppressed**			
**Gene Name**	**Description**	**Inhibitor**	**Expression**

ARL11	ADP-ribosylation factor-like 11	Ly294002	U
B2M	beta-2-microglobulin	Ly294002	U
CECR1	cat eye syndrome chromosome region, candidate 1, transcript variant 1	Ly294002	U
PCDH10	protocadherin 10, transcript variant 1	Ly294002	U
PCDH11Y	protocadherin 11 Y-linked, transcript variant c	Ly294002	U
PPP1R12B	protein phosphatase 1, regulatory (inhibitor) subunit 12B, transcript variant 3	Ly294002	U
PSORS1C1	psoriasis susceptibility 1 candidate 1	Ly294002	U
ST3GAL6	ST3 beta-galactoside alpha-2,3-sialyltransferase 6	Ly294002 rapamycin	U
CDKN2B	cyclin-dependent kinase inhibitor 2B (p15, inhibits CDK4), transcript variant 1	rapamycin	U

**MCF-7 breast cancer cell line, RPS6KB1-suppressed**			

**Gene Name**	**Description**	**Inhibitor**	**Expression**

COL5A1	collagen, type V, alpha 1	Ly294002	U
FAM5C	family with sequence similarity 5, member C	Ly294002	U
RELB	v-rel reticuloendotheliosis viral oncogene homolog B, nuclear factor of kappa light polypeptide gene enhancer in B-cells 3 (avian)	Ly294002	U
SOX15	SRY (sex determining region Y)-box 15	Ly294002	U
ST3GAL6	ST3 beta-galactoside alpha-2,3-sialyltransferase 6	Ly294002	U
VTCN1	V-set domain containing T cell activation inhibitor 1	Ly294002	D
IFI44L	interferon-induced protein 44-like	rapamycin	D
OASL	2'-5'-oligoadenylate synthetase-like, transcript variant 1	rapamycin	D
SCD	stearoyl-CoA desaturase (delta-9-desaturase)	rapamycin	D

The genes were differentially expressed to the same direction by small molecule inhibitors and at least two *RPS6KB1 *siRNAs.

Abbreviations: D = downregulated expression, U = upregulated expression.

### Gene ontology (GO) analysis and Connectivity Map support the known effects of Ly294002 and rapamycin as well as suggest new inhibitors with similar mechanism of action

We then explored which gene ontology classes were enriched in gene expression profiles of five Ly294002- and rapamycin-treated breast cancer cell lines [see Additional File 5] by using Gene Ontology Categorizer [17]. Among the twenty relatively most enriched GO classes in Ly294002-treated cell lines were functional categories involved in cell killing, mitosis, and G1 phase of the cell cycle. In rapamycin treatment, these included functional categories such as mitosis, M phase of mitotic cell cycle and translational elongation. To identify other clinically approved drugs with potentially similar mechanisms of action, we took advantage of the recently published Connectivity Map [18], where connections of chemical or biological perturbations can be identified using a web-based interface. As expected, Ly294002 gave the highest positive connectivity for Ly294002-treated samples. Similarly, rapamycin gave a high positive connectivity with fairly low p-values (<0.059) for rapamycin-treated samples. The novel drugs with high positive connectivity with our Ly294002- and rapamycin-treated gene expression profiles included wortmannin, trichostatin A, and rottlerin.

## Discussion

The 17q23 region is one of the most highly amplified regions in breast cancer and *RPS6KB1 *is considered one of its target genes [4,19]. Due to *RPS6KB1 *amplification and overexpression in breast cancer and the role of p70S6K as a downstream mediator of PI3K/mTOR pathway, our aim was to identify PI3K/mTOR/p70S6K pathway downstream targets using gene expression profiling for breast cancer cell lines that we have previously characterized in regard to copy number and gene expression [4,19]. Five breast cancer cell lines were treated with PI3K/mTOR pathway inhibitors, including two cell lines that were also inhibited with three *RPS6KB1 *siRNAs, since these two cell lines show a high-level expression of p70S6K. The gene expression signatures responsive to both PI3K/mTOR pathway and p70S6K inhibitions revealed previously unidentified genes suggesting novel downstream targets for PI3K/mTOR/p70S6K pathway.

The PI3K pathway is deregulated in a number of cancers and clinical trials are currently attempting to target different components of this pathway [12]. The activation of PI3K pathway is associated with aggressive breast cancer and therefore, identification of its downstream targets may have diagnostic value [20]. Since p70S6K is a downstream mediator of PI3K pathway, we explored gene expression alterations caused by both p70S6K and PI3K/mTOR pathway inhibitions. Altogether, expression levels of 17 genes were altered in both *RPS6KB1*-suppressed and inhibitor-treated samples, including *VTCN1/B7-H4*, *CDKN2B*, *SCD *and *ARL11*. Especially, the down-regulation of *VTCN1*/*B*7-*H*4 is interesting, since it has recently been found to be overexpressed in a number of cancers and have also been linked to poor prognosis [21,22]. The expression of B7-H1, which is a member of the same protein family, has been shown to be increased due to loss of *PTEN *and activation of PI3K pathway, linking PTEN with immunoresistance mediated in part by B7-H1 [23]. The down-regulation of *VTCN1*/*B*7-*H*4 due to Ly294002 treatment suggests that similar to B7-H1, B7-H4 is also regulated by PI3K. *CDKN2B*, encoding for cyclin-dependent kinase inhibitor 2B, was up-regulated after mTOR and p70S6K inhibitions in BT-474. Similarly, *CDKN2A*, was up-regulated in MCF-7 in response to p70S6K suppression but not with inhibition of mTOR. The p16/p15 proteins encoded by *CDKN2A*/*CDKN2B *are known to be associated with telomerase activation and cancer progression [24,25] as well as to induce cell cycle arrest by inhibition of CDK4 kinase [26]. Interestingly, mTOR inhibition caused significant down-regulation of *CCND1 *suggesting that cell cycle arrest could be caused by either down- or up-regulation of genes activating or inhibiting CDK4. Additionally, *SCD *was down-regulated after mTOR inhibition with rapamycin and p70S6K suppression with *RPS6KB1 *siRNAs. *SCD *encodes stearoyl-CoA desaturase, which has been suggested to promote cell proliferation, invasion and inhibition of apoptosis [27]. Our finding of *SCD *as a potential downstream target of PI3K/mTOR/p70S6K pathway is supported by the study of Chang *et al*., which reported that *SCD *is activated by PI3K and inhibited by Ly294002 [28]. Most of the genes identified in our study did not have any previous interaction with PI3K/mTOR/p70S6K pathway. For example, *ARL11*, a gene with recently identified tumor suppressor function [29], was up-regulated after both inhibiting PI3K and suppression of p70S6K. Taken together, our study suggests many novel targets potentially regulated by PI3K/mTOR/p70S6K pathway.

Overall, the number of overlapping genes between three treatments was small, which is expected, since all the inhibitors have different targets with cross-talk also with multiple other pathways. In addition to these biological reasons, there are technical differences that are likely to cause variability for gene expression changes caused by different inhibitors. These include different specificities, efficiencies and mechanisms of action of the treatments as well as differences in assay platforms. For example, Ly294002 is not exclusively selective for the PI3Ks, but is known to inhibit also other lipid kinases than just PI3K [30]. Secondly, possible off-target effects of *RPS6KB1 *siRNA knock-down or the fact that knock-down mainly resulted in 70% decrease in mRNA levels are possible reasons for variability between different experiments. Finally, inhibitor-treated samples were studied using a 17 K gene expression microarray platform, whereas siRNA-treated samples were hybridized on a 44 K microarray platform causing slight differences in the gene content of these two microarray platforms. To minimize the above-mentioned effects, we took into account only those genes that were differentially expressed by at least two different siRNAs and correlated this information with genes responsive to PI3K and/or mTOR inhibition. This led to the identification of 17 genes that are potential targets of PI3K/mTOR/p70S6K pathway.

Since PI3K/mTOR pathway has a central role in cell survival and p70S6 kinase is an important regulator of cell cycle progression through the G1/S point, we further studied the effect of Ly294002 and rapamycin on apoptosis and cell cycle. Ly294002 treatment, but not rapamycin, induced apoptosis of the breast cancer cell lines, which may be due to a number of different reasons. Ly294002 inhibits PI3K that further regulates AKT, which has a number of targets that are involved in cell death and survival [5]. In contrast, rapamycin inhibits mTOR, which is located downstream of AKT and therefore, AKT mediated effects on apoptosis should not be detected after rapamycin treatment. Additionally, mTOR has been shown to be involved in Notch1-mediated inhibition of p53-induced apoptosis [31]. Since the majority of the breast cancer cell lines used in this study carry a mutated form of p53 [32-35], inhibition of mTOR by rapamycin should not affect on p53-induced cell death. Rapamycin also inhibits the phosphorylation and activation of p70S6K through mTOR inhibition. It has been observed that the breast cancer cell lines with an amplification of *RPS6KB1 *are more sensitive to rapamycin than cells with no amplification [36]. Indeed, also in our study, the breast cancer cell lines BT-474, MCF-7 and MDA-361, which have an amplification of *RPS6KB1*, seemed to be sensitive to the rapamycin-induced G1 phase arrest. MCF-7 and BT-474 breast cancer cell lines have mutated forms of *PIK3CA *[37,38], which can render these cells resistant to Ly294002-mediated cell cycle arrest. Surprisingly, Ly294002 treatment resulted in G1 arrest in MCF-7 cells in addition to SK-BR-3 cell line. This discrepancy might be explained by the possibility that MCF-7 cell line harbor mutation with no activating function of PI3K. In contrast, no arrest was detected in BT-474, MDA-361 and MDA-436 cells. Taken together, our results confirm the previously observed effects of these inhibitors on cell cycle and suggest that different breast cancer cell lines have different biological responses to PI3K/mTOR pathway inhibitors; especially MDA-436 seems to be resistant to rapamycin- and Ly294002-induced cell cycle arrest.

The inhibition of PI3K/mTOR pathway with Ly294002 and rapamycin led to similar gene expression alterations in different breast cancer cell lines. Altogether, 38% of the differentially expressed genes were altered by both treatments. Additionally, a number of genes known to be associated with PI3K/mTOR pathway were differentially expressed. For example, the down-regulation of eIF4G1 in response to rapamycin and Ly294002 treatments was also shown at protein level. This suggests that PI3K/mTOR pathway inhibition leads to the transcriptional deregulation of a number of critical components of the translational machinery. Unlike with PI3K or mTOR inhibition, direct suppression of p70S6K did not seem to down-regulate genes involved in eIF-4F initiation complex. This might be due to the fact that p70S6K is known to regulate the rate of translation of transcripts encoding elongation factors and ribosomal proteins [6], but inhibition of p70S6K do not affect on transcriptional activation of these genes. Gene Ontology Categorizer [17] and recently published Connectivity Map [18] were further used to explore the biological processes affected by PI3K/mTOR pathway inhibition and drugs with similar mechanism of action. Indeed, GO categories involved in cell killing, mitosis, and G1 phase of the cell cycle were enriched in Ly294002-treated cells, whereas functional categories like mitosis and translational elongation were among the most enriched classes with lowest p-values in rapamycin-treated cells. Also Connectivity Map gave the highest scores for Ly294002 and rapamycin in the breast cancer cell lines treated with these inhibitors further validating the gene expression profiles responsive to these PI3K and mTOR inhibitors. Also wortmannin scored high in Connectivity Map, which is expected, due to its mechanism as a PI3K inhibitor. Other drugs with high statistical significance included rottlerin, a protein kinase inhibitor, and trichostatin A, a known HDAC inhibitor, both of which are known to inhibit proteins interacting with PI3K/mTOR pathway.

A number of studies have tried to find markers for pathway activation, since activation of PI3K/AKT/mTOR pathway is known to be associated with aggressive cancer. Cancer drugs are increasingly designed to target specific signaling pathways and also in this regard microarrays have been used to identify oncogenic signatures aiming to determine the activation state of specific pathways [39]. Recently, Saal *et al*. identified a new marker stathmin (*STMN1*) to be associated with PTEN mutation and PI3K activation in breast cancer. Stathmin was also found to be down-regulated due to Ly294002 treatment [20] that is in line with our data [see Additional File 2]. Recently, a transcriptional signature specific for AKT1 activation and subsequent mTOR inhibitor RAD001 treatment was identified in luminal epithelial cells of the mouse ventral prostate [40]. In another recent study, the presence of this transcriptional signature was evaluated in five publicly available microarray data sets from clinical breast tumors [41]. Altogether, 57 AKT1-signature genes had p-values less than 0.01 in three breast cancer data sets, from which 34 genes were regarded as RAD001-insensitive and 23 genes as RAD001-sensitive. We also evaluated whether these genes would be differentially expressed in response to rapamycin treatment in our study. Interestingly, 21% (7/34) of the genes that positively correlated with *AKT1 *expression in a study by Creighton and co-workers, correlated positively with *AKT1 *expression also in our rapamycin-treated breast cancer cell lines. However, in Creighton's study, the expression of these genes did not change due to RAD001 treatment and therefore, these genes were considered RAD001-insensitive. In our data, these genes were down-regulated due to rapamycin treatment opposite to the observation in clinical breast tumors, in which these genes were up-regulated together with *AKT1*. These results support the idea that these genes are co-expressed with *AKT1*, although based on our data their role in rapamycin sensitivity could not be confirmed.

In the present study, we took the approach to assess transcriptional alterations in response to inhibition of PI3K/mTOR/p70S6K pathway in breast cancer cell lines with known gene copy number and gene expression alterations, since *RPS6KB1 *encoding p70S6K is one of the most highly amplified and overexpressed genes in breast cancer. The inhibition of PI3K/mTOR pathway by small molecule inhibitors led to similar gene expression alterations across several breast cancer cell lines with different biological outcomes. Since no specific inhibitor for p70S6K is currently available, we prompted to use three different *RPS6KB1 *siRNAs for inhibition of p70S6K in cell lines with high-level amplification and overexpression of *RPS6KB1*. Altogether, 109 and 173 genes were differentially expressed in two *RPS6KB1*-suppressed breast cancer cell lines, including 68 genes of which expression was altered in both cell lines and 17 genes that overlapped with genes differentially expressed after the small molecule inhibitor treatments. We suggest that these 17 genes that were differentially expressed after both PI3K or mTOR inhibitions and p70S6K inhibition with at least two siRNAs against *RPS6KB1 *are potential downstream targets of PI3K/mTOR/p70S6K pathway.

## Conclusion

We identified a number of genes that were differentially expressed due to p70S6K suppression and PI3K/mTOR pathway inhibitions suggesting novel downstream targets of PI3K/mTOR/p70S6K pathway. These include *VTCN1 *and *CDKN2B*, whose functional role in breast cancer downstream of this pathway requires further investigation. Due to the association of p70S6K overexpression with aggressive disease and poor prognosis of breast cancer patients, the downstream targets of p70S6K may have diagnostic value.

## Methods

### Suppression of RPS6KB1 expression by siRNAs

BT-474 and MCF-7 breast cancer cell lines with high-level amplification and overexpression of *RPS6KB1 *[4] were transfected with three synthetic siRNAs targeting *RPS6KB1 *[GenBank: NM_003161]: siRNA 1 (siRNA ID#: 1454, sense strand: 5'-GGACAUGGCAGGAGUGUUUtt-3', antisense strand: 5'-AAACACUCCUGCCAUGUCCtc-3'), siRNA 2 (siRNA ID#: 142750, sense strand: 5'-GCUACUUCGGGUACUUGGUtt-3', antisense strand: 5'-ACCAAGUACCCGAAGUAGCtc-3') and siRNA 3 (siRNA ID#: 110802): sense strand 5'-GGUCAUGUGAAACUAACAGtt-3', antisense strand 5'-CUGUUAGUUUCACAUGACCtt-3') purchased from Ambion^® ^(Austin, TX). Transfections were performed using the "Transfecting Stealth™ RNA or siRNA into Mammalian Cells Using Lipofectamine™ 2000" protocol according to the manufacturer's recommendations (Invitrogen, Carlsbad, CA). 150,000 cells per well were plated in 2.5 ml of culture medium onto a 6-well plate 24 hours before the siRNA transfections. For BT-474 cell line, 7.5 μl of Lipofectamine2000 transfection reagent (Invitrogen) and 50 pmol of *RPS6KB1 *siRNA were used. For MCF-7 cell line, the conditions were 7.5 μl of Lipofectamine2000 and 200 pmol of siRNA. After four hours of transfection, the cell culture medium was replaced with fresh medium and the cells were incubated altogether for 72 hours. The cells from two parallel wells were pooled and the total RNAs of BT-474 and MCF-7 breast cancer cell lines were isolated using Qiagen RNeasy Mini Kit (QIAGEN, Valencia, CA). The quality of the RNA was assessed by 2100 Bioanalyzer (Agilent Technologies, Palo Alto, CA) to control the integrity of the samples before microarray hybridizations.

### Inhibition of PI3K/mTOR pathway by small molecule inhibitors

Five breast cancer cell lines, BT-474, MCF-7, MDA-MB-361, MDA-MB-436, and SK-BR-3, were treated with 50 μM of PI3K inhibitor Ly294002 (Cell Signaling Technology, Danvers, MA) and 100 nM of mTOR inhibitor rapamycin (Calbiochem, Darmstadt, Germany) and the cells were harvested at 24 h and 48 h time points. The cell lines were purchased from ATCC and cultured according to the recommended conditions. The RNA was extracted using TRIzol^® ^reagent (Invitrogen, Carlsbad, CA) according to the manufacturer's instructions. After the Trizol extraction, the RNAs were purified using Qiagen's (Valencia, CA) RNeasy column purification to remove remnants from Trizol extraction. Then, all the RNAs were run by 2100 Bioanalyzer (Agilent) before microarray hybridizations.

### Protein assays by Western immunoblotting

Western immunoblotting was carried out to study the protein levels of p70S6K, AKT, mTOR and eIF4G1 after the perturbation of the breast cancer cells with PI3K/mTOR pathway inhibitors or *RPS6KB1 *siRNAs. Additionally, phosphorylation of p70S6K was determined in Ly294002- and rapamycin-treated cells to study whether the inhibitors blocked the phosphorylation of p70S6K Thr389. The inhibitor- and siRNA-treated cells harvested at the time points were lysed with 150 μl of boiling hot SDS lysis buffer (2.5 ml 20% SDS, 5 ml 1 M Tris pH 6.8, 12.5 ml dH_2_O) per well on a 6-well plate. The downregulation of p70S6K was detected with rabbit p70S6 kinase antibody (Santa Cruz Biotechnology, Santa Cruz, CA) in 1:1000 dilution and rabbit β-actin antibody (1:1000, Cell Signaling, Danvers, MA) was used to detect the equal loading of samples. The effect of the inhibitors on p70S6K phosphorylation was studied using rabbit p-p70S6 kinase antibody for Thr389 phosphorylation (Santa Cruz Biotechnology) in 1:1000 dilution. The total protein amount of p70S6 kinase was studied as above. We also performed protein-level validation of the microarray results with the following antibodies: Akt (1:1000, Cell Signaling), mTOR (1:500, Cell Signaling), and eIF4G1 (1:500, Abcam, Cambridge, UK). The detection of the proteins was performed using anti-rabbit secondary antibody (1:1000, Amersham Biosciences, Pittsburgh, PA) and chemiluminescence by ECL detection kit (Amersham Biosciences).

### Apoptosis and cell cycle assays

Apoptosis and cell cycle assays were performed for inhibitor-treated breast cancer cell lines and their controls to evaluate biological response to PI3K/mTOR pathway inhibitors. To study whether the inhibitors or *RPS6KB1 *siRNAs induced apoptosis, caspase-3 activity was measured from 30 or 40 μg of protein from each inhibitor- and siRNA-treated cell lines using colorimetric caspase-3 activity (Ac-DEVD-pNa cleavage) assay as described previously [42]. Additionally, the morphology of the cells was evaluated under the light microscope to determine the number of apoptotic cells in inhibitor-treated cells as compared to the untreated controls. For the cell cycle assays, cells were grown in duplicates on 96-well cell culture plates and harvested at 24 h and 48 h time points. Trypsinized cells were centrifuged 200 × g for 3 min and then treated with 170 μl of cold 70% ethanol followed by incubation o/n in -20°C. After the incubation, the cells were centrifuged for 3 min, the supernatant was removed and the cells were stained with 80 μl of RNase A/propidium iodide in PBS (30 μg/ml). The cells were then incubated at +37°C for 45 min and stored at +4°C until they were analyzed using BD FACSArray Bioanalyzer System (BD Biosciences, San Jose, CA). An average of 15,000 events was analyzed per each well.

### Gene expression analysis by oligonucleotide microarrays

Microarray analyses from inhibitor-treated cell lines were performed using Agilent's Human 1A Oligo Microarrays containing 17,986 genes or transcripts. The inhibitor-treated samples were hybridized against the corresponding untreated cell line harvested at 24 h and 48 h time points. The labeling was performed from 20 μg of total RNA using direct labeling method according to the manufacturer's instructions (Agilent Technologies). The RNAs extracted from the *RPS6KB1 *siRNA-suppressed BT-474 and MCF-7 cell lines were hybridized against their corresponding control cell lines transfected with siRNA Scramble Duplex (Dharmacon Research Inc., Boulder, CO) using identical conditions as with *RPS6KB1 *siRNA. Five-hundred nanograms of total RNA was labeled according to the manufacturer's recommendations (Agilent Technologies). The RNAs were hybridized on Agilent's Human 4x44K Oligo Microarrays containing 45,220 features. Two technical replicates were performed for each hybridization and the logarithmic transformed (base 10) mean gene expression ratios were taken for further analysis. Pre-processing of the data was performed by Feature Extraction software (v 6.1.1 and 9.5.3.1 for inhibitor- and siRNA-treated samples, respectively). Only those genes, for which the mean signal log 10 ratio from inhibitor-treated cell lines or the two siRNA-suppressed replicates was ≥ 0.3 or ≤ -0.3 with p values ≤ 0.05 representing two-fold up- or down-regulation, respectively, were considered differentially expressed. The data from the inhibitor-treated and the siRNA-transfected samples were analyzed separately due to different processing of the samples. All the raw data is available at CanGEM (please see Availability & requirements for more information).

### SOM (self-organizing map) clustering of inhibitor-responsive gene expression signatures

To further study the genes that were identified as differentially expressed due to PI3K/mTOR pathway inhibition across all treated breast cancer cell lines, SOM clustering algorithm with component plane presentation was used to analyze and visualize the differences between the drug-treated cell lines [43,44]. Only the differentially expressed genes in each inhibitor treatment, whose standard deviation (SD) was >3 in at least one of the samples, were included into the clustering step. The SOM Toolbox for MATLAB [45] was used with the following parameters: sheet SOM map topology with batch learning, Euclidean distance and Gaussian neighborhood function.

### Gene Ontology mapping of inhibitor-responsive gene expression profiles

To highlight functionally important biological responses to PI3K/mTOR pathway inhibitors, the representation of gene ontology classes among differentially expressed genes (SD>3) in inhibitor-treated breast cancer cell lines was explored using The Gene Ontology Categorizer [17]. First, the updated Ensembl IDs (please see Availability & requirements for more information) were retrieved for all the genes with SD>3 among rapamycin and Ly294002 treatments. The GO classes were first sorted by their relative enrichment. Twenty most enriched GO classes were then sorted according to their p-values of relative enrichment [17].

### Similarity search of inhibitor-induced gene expression profiles by Connectivity Map

To study whether other small molecules would cause similar transcriptional alterations in human cell lines, the inhibitor-perturbed gene expression data was downloaded into the Connectivity Map, which is a web-based catalogue of gene expression data from chemically treated cultured human cells [18]. The Agilent probe IDs were first transformed into Affymetrix probe IDs using Ensembl (please see Availability & requirements for more information). The gene lists containing a maximum of 1000 up- and downregulated genes were loaded into the Connectivity Map (please see Availability & requirements for more information). The drugs giving the highest scores for similarity with rapamycin- or Ly294002-treated breast cancer cells were regarded as inhibitors with similar mechanisms of action.

## Abbreviations

GO: gene ontology; mTOR: mammalian target of rapamycin; p70S6K: 70 kDa ribosomal protein S6 kinase; PI3K: phosphoinositide-3-kinase; SOM: self-organizing map

## Availability & requirements

CanGEM: 

Ensembl: 

Broad Institute, Connectivity Map: 

## Authors' contributions

OM designed and supervised the study. HH performed the RNAi experiments, inhibitor treatments and Western assays. AN and JK carried out the apoptosis analyses. HH, MS and SH carried out the bioinformatics analysis of the microarray data. AK was involved in setting up the study. HH and OM wrote the manuscript. All the authors have read and accepted the final version of the manuscript.

## Supplementary Material

Additional File 1 Differentially expressed genes after *RPS6KB1 *siRNA treatments in breast cancer cell lines. Click here for file

Additional File 2 Differentially expressed genes after inhibitor treatments in breast cancer cell lines. Click here for file

Additional File 3 Effects of Ly294002 and rapamycin treatments on cell cycle arrest in breast cancer cell lines. Click here for file

Additional File 4 Common differentially expressed genes by inhibitor and siRNA treatments in BT-474 and MCF-7. Click here for file

Additional File 5 Enriched gene ontology (GO) classes after Ly294002 and rapamycin treatments in breast cancer cell lines. Click here for file
